# Socioeconomic status and sleep disturbances among pediatric population: a continental systematic review of empirical research

**DOI:** 10.5935/1984-0063.20200082

**Published:** 2021

**Authors:** FA Etindele Sosso, Tommy Khoury

**Affiliations:** 1 Department on Global Health and Ecoepidemiology,Redavi Institute, Montréal, Canada.; 2 Université de Montréal, Faculté de Médecine - Montréal - Québec - Canada.

**Keywords:** Adolescent, Sleep, Child, Social Class, Socioeconomic Factors, Systematic

## Abstract

To this day, no consensus has been established on the definition and the conceptualization of the socioeconomic status (SES), since all the available studies on the relation between SES and health did not use the same conceptual framework and operationalization to assess SES. While literature reported that SES markers (such as income, social support networks, education, employment or occupation) influence the health of populations by shaping living conditions; empirical research does not tell us which SES markers affect more strongly the sleep components of the individuals, as well as which sleep disorders (SD) are affected and how. Even though several original studies have tried to assess how changes in socioeconomic status of parents may affect the psychosocial environment and mental health of an individual directly or through his community, no systematic reviews on the influence of SES on children’s sleep are available. This systematic review make an update on the different measures of SES and sleep disturbances used for pediatric population across the different regions of the world. Recommendations for a future standardization of SES measures is proposed, for a better understanding of its influence on sleep disturbances.

## INTRODUCTION

It has now become a documented reality that socioeconomic status (SES) has an important association with negative changes in global health^1,2^. Even if SES is not measurable or observable directly, many indicators of its effects exist and can be used to assess its influence on health^3,4^. The social determinants of health, such as income, social support networks, education, employment or occupation, influence the health of populations by shaping living conditions^3-5^. These living conditions in turn determine how an individual handles his health, with different patterns of health outcomes appearing through time, as documented by epidemiological studies. Literature demonstrated that people with high education and income, like white-collar workers, reported fewer chronic diseases than those with physical occupations like blue-collar workers^6-8^. Furthermore, people with higher levels of education and higher incomes have a lower prevalence of sleep disorders, mood disorders, musculoskeletal impairment, and chronic diseases, such as diabetes, morbid obesity, or cardiovascular diseases^6-8^. 

Despite the important epidemiological input, which showed a negative association between SES markers, like low income, education or neighborhood, with decrease of health, sleep is poorly studied. Sleep is a physiological function that may be affected by multiple biological, psychological and environmental factors^9-12^. Few studies have found association between sleep disorders of individuals with work and family characteristics^13-16^, race or ethnicity^17-20^ and occupation or shift-work^21,22^. To this day, no consensus has been established on the definition and the conceptualization of SES, since all the available studies on the relation between SES and sleep do not use the same indicators to assess SES. One reason is the conceptualization of SES, which is different from a world region to another, which in turn influences the measurement/operationalization of this same SES through the studies. Another reason is the fact that some sleep disorders like insomnia, are more studied than others like restless legs syndrome or nightmares^22^. Currently, empirical research does not tell us which SES markers more strongly affect the sleep components of the individuals, as well as which sleep disorders are affected and how. Data also does not document during which time this effect takes place, and at which part of the human life the influence of SES starts to be deleterious.

Another important point is the huge number of studies devoted to adults and older people, compared with those investigating effects of SES on children and adolescents sleep^22-25^. Recently, accumulating evidence on social determinants of health revealed that a socioeconomic gradient of health affects mental health of adults, and negative changes related to this influence may start earlier than past literature hypothesized^26^. By studying the association of SES indicators, such as education, income, household, and occupation, with presence of sleep disturbances, it will be easier to understand how SES is linked to sleep disorders for the general population of adults and the elderly. Even though several original studies have tried to assess how changes in socioeconomic status of parents may affect the psychosocial environment and mental health of an individual directly or through his community^18,20,27-33^, no systematic reviews on the influence of SES on children’s sleep are available. 

The aims of this systematic review are: 1) to provide a picture of the research activity on this relation in each world region; and (2) to assess the relation between SES and sleep disorders in the general population of children and adolescents.

## MATERIAL AND METHODS

### Literature search

**Figure 1:**
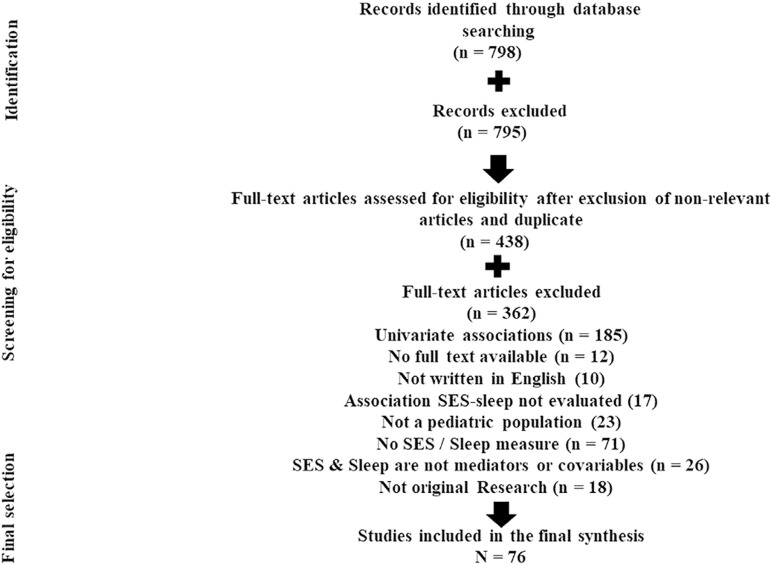
Prisma flowchart.

A search in PubMed/MEDLINE and Google scholar was performed to identify relevant articles investigating the relation between SES and various sleep indices following the PRISMA guidelines (Figure 1). The search strategy included the following keywords: socioeconomic* OR socio-economic* OR “social status” OR “social position*” OR “social class*” OR “social rank*” OR education* OR income* OR occupation* OR employment OR *employed OR asset* AND sleep* OR insomnia* OR circadian OR parasomnia* OR “restless leg*” OR “periodic leg movement* AND “adolescent” OR “children*” OR child* OR childhood* OR youth* OR youthness*” OR “infant*”. The search strategy was restricted to published articles from January 1990 to September 2019 and excluded articles that did not perform any qualitative or quantitative investigation such as reviews or meta-analyses, commentary, opinion, editorial, and proceedings of congress.

### Inclusion and exclusion criteria

Observational studies investigating sleep of children and adolescents aged until 19 years old were included. There is no restriction of sex, SES measures, type of sleep disorders, and race/ethnicity from the general population of children and adolescents. All objective SES markers (such as occupation, household income or material deprivation) and subjective SES markers (perceived indicators) were considered for the individuals, their parents, or the family. We evaluated several objective sleep components such as wake after sleep onset (WASO), total sleep time (TST), sleep efficiency (SE), sleep latency (SL), and apnea-hypopnea index (AHI). We also considered subjective reports about sleep quality, sleep duration and every composite measure of sleep. Self-reported symptoms of sleep disorders were collected from interviews, questionnaires, and surveys. Objective tests included sleep diaries, wrist actigraphy, and polysomnography (PSG).

The articles excluded of the final synthesis were: 1) interventional trials, reviews, meta-analyses, case series or case reports, and articles not presenting original results; 2) written in another language than English; 3) not accessible in full text; 4) included participants with any disease related directly or indirectly with sleep disorders; 5) only reporting univariate associations and unadjusted estimates of the variables of interest (SES and sleep).

### Literature synthesis

The information extracted from chosen studies were setting, population, age of participants, SES measures, sleep measures, and main outcomes. For each comparison between SES measures and sleep outcomes, the quantity of articles that assessed them was reported. In this article, results are presented separately by continents or regions (North America, Latin America, Europe, Africa, Asia, and Oceania), to see which conceptualization of SES and findings related to children’s sleep are developed for each region.

## RESULTS

A total of 76 published articles were included in the final synthesis, the oldest published in 1997 and the latest in 2019. From these studies, twenty-seven were performed in North America, six in South and Central America, sixteen in Europe, four in Oceania, twenty-one in Asia and one in Africa. There is also 1 multinational study performed simultaneously in 12 countries (Australia, Brazil, Canada, China, Colombia, Finland, India, Kenya, Portugal, South Africa, the United Kingdom, and the United States of America). Most of questionnaires used to assess sleep disturbances were applied or filled by parents. Population age ranged from birth until nineteen years old.

## NORTH AMERICA

Among the twenty-seven articles published in North America on this topic, twenty four were made in USA^20,28,34-46^ and three were performed in Canada^47-49^. Among these studies, eighteen were cross-sectional studies^28,35,36,40-45,49-57^ and the rest were longitudinal cohorts^20,34,37-39,46-48,58^. The participant age ranged from 1 yr.^52^ to 19 yrs.^41,54^. Several different measures of SES were used across time and studies regardless of study design or sleep disorders assessed. Often it is only one SES indicator used for the study, while sometimes, a combination of markers is used, or a questionnaire/scale for subjective measures. The following measures were used from 1997 to 2019: *the financial need* (estimated with the family’s participation to the school lunch assistance program)^50^, *the family’s self-reported standard of living* (reported by individuals or their parents as: very well off, living comfortably, just getting along, nearly poor or poor)^51^, *the Hollingshead score* (a survey designed to measure social status of an individual or that of his parents based on the following domains: marital status, retired/employed status, educational attainment, and occupational prestige)^20,52^*, the parental income* (median household income, annual income, median annual income, family economy)^39,41,44,49,53-55,57,58^, *the education* (parental education, individual’s level of education, average parents’ years of education, highest diplomas and maternal education)^36,37,39,40,42,44-46,54,57^, *the family economic hardship* (it is a composite score of a 17-item questionnaire measuring inability to make ends meet, not having enough money for necessities, economic adjustments or cutbacks, financial strain)^56^, *the ratio of income to poverty* (the total family income divided by the poverty threshold is called the “ratio of income to poverty”)^36^, *the income-to-needs ratio* (computed by dividing family income by the federal poverty threshold for that family size)^34,35,38,43,45^, *the composite measures or indexes* (calculated with a combination of SES indicators like education, income, area of living, etc.), which are the main measures of SES available in literature^34,37,40,44,47,49^, *the subjective childhood SES* measured with the MacArthur Scale (this scale allows a self-report of perceived SES using the general socioeconomic markers such as income, occupation and education)^28^, and *the ethnicity/race* which is generally used in the conceptualization of SES in USA, and mainly used to dichotomize population in subgroups of high SES (Caucasian people) and low SES (it is often non Caucasian American communities)^20,45,53^.

There are several conceptualizations of SES leading to the variety of measures above. For sleep in those studies, it is more precise and conventional. Sleep measures are generally classified as objective and subjective, depending on the instruments used by the researcher. Literature from 1990 to now, reported data collected with the following type of measures: *the questionnaires/scales* [the pediatric sleep questionnaire^50,59^, the sleep/wake problems scale (also named the child-reported sleep/wake problems scale)^43,45^, the pediatric daytime sleepiness scale^49^, the children’s sleep habits questionnaire^47,49,60^, the Pittsburgh sleep quality index^28,41^, the sleep-wake problems behavior scale^35^ and the Epworth sleepiness scale-revised for children^35^), *the actigraphy* which is an objective measure of sleep parameters (sleep start time, sleep end time, sleep period time, sleep time, wake time, wake bouts, sleep efficiency, longest continuous sleep)^20,34,43,45,52,57^ and *the self-reported items* (by parents most of the time) measuring subjective symptoms of sleep disorders, based on DSM criteria, ICD criteria, or embedded in a non-specific questionnaire (and non-validated) used in national survey and longitudinal cohort^20,36-40,42,44,46,48,51,53-56,58^. This kind of sleep measure is widely used in the studies investigating effects of SES on children sleep until now.

When looking at these studies altogether, some key findings in North American literature seem to be emerging on the influence of SES on children’s sleep. Poor subjective childhood SES was associated with poor sleep quality^28^. The association was moderated by risky family environment because in less risky family environments, childhood SES was not associated with sleep quality^28^. Higher early SES was associated with longer sleep duration, shorter sleep latency and lower midpoint variability, with a mediation role played by the quality of the home environment^34^. Even if environmental and behavioural characteristics (maternal smoking status, child TV/video viewing, active play, breastfeeding, presleep worries) partially mediated the relationships between SES and sleep^39,43,61-63^, poor standards of living and lifestyle predicted insomnia diagnosis^51^ and sleep inadequacy^40^. 

The area of living and the neighborhood plays an important role because sufficient sleep was more likely among students attending schools in areas classified in the highest SES group while attending schools in low-income areas predicted short and low normal sleep duration trajectories over time^48^. Children with a lower family SES woke up later in the morning, spent longer times in bed, and had more nocturnal wake time, more wake bouts, shorter continuous sleep bouts, and lower sleep efficiency^52^. Children in families with lower SES had more night- time variability in bedtimes, sleep start times, and sleep period times^52^. Low SES children were more likely to have a shorter sleep duration, problematic bedtime behaviour, and EDS (excessive daytime sleepiness)^53^ and reported to have more sleep behaviour problems than the higher SES children^53^. 

Lower SES predicted sleep rhythmic movements in children^47^ and greater WASO was associated with lower baseline childhood SES, decreasing childhood SES and lower adult SES^20^. In children, higher subjective SES predicted less daytime sleepiness and longer self-reported sleep duration and higher household income predicted longer parent-reported sleep duration^49^. In adolescents, higher subjective SES was associated with better sleep quality, while shorter sleep duration and higher household income was associated with fewer sleep disturbances^49^. Regarding income, lower income-to-needs ratio predicted higher levels of reported sleep/wake problems. African American children’s sleep was more negatively affected by income-to-needs ratio than was the sleep of European American children^45^. Lower income-to-needs ratio was also related to greater sleep/wake problems and increased sleepiness^43^; whereas the improvement in family income-to-needs is associated with longer sleep for children and adolescents^38^. Lower parental perceived economic well-being was associated with shorter sleep minutes and greater variability in sleep onset^45^. Higher income quartile was associated with less frequent diagnosis of sleep disorders in preschool and school-aged children and adolescents^55^. Concerning education, higher parental education was associated with shorter sleep duration^56^. Higher parental education was associated with higher odds of sufficient sleep duration^42^. Lower maternal education was associated with lower sleep efficiency and higher chronic sleep curtailment in American children^39,45^. Students whose parents had completed some college education compared to those with high school or less reported greater weekday sleep insufficiency^37^. Adolescents whose highest degree was over a high school degree were less likely to have sleep problems compared with young adults whose highest degree was a community college credential^46^. 

Regarding the ethnicity, which is widely used in social epidemiological research related to health inequalities or socioeconomic disparities in USA, African American and non-Caucasian people were always considered in the lower SES group (regardless of their education, area of living and neighborhood). Older African American females in the lower SES group were reported to obtain the least sleep, while older Caucasian males in the upper SES group were reported to obtain the most sleep^53^. Shorter sleep duration was associated with increasing childhood SES only in the white population^20^, while lower mother’s education was associated with more chronic sleep disturbances in African American children and non-Caucasian American children compared with European American children^39,45^. 

Finally, regarding employment influence on children’s sleep, children whose mothers worked were more likely to have a lower amount of sleep at night^36^, while children whose mothers were employed full-time were less likely to sleep longer hours compared to those whose mothers were employed <20h per week^44^

### Latin America

Literature on the influence of SES on children and adolescent sleep in South and Central America is interestingly dominated by Brazil, where the six studies included in the PRISMA search originate^64-69^. The participant age ranged from 3-month-old new-borns^65^ to 19 year-old adolescents^64,69^. These studies were published between 2013 and 2018. For their conceptualization of SES, researchers used the three following indicators as main measures: *the income* (primarily family income and household income)^64-66,68,69^, *the education* (maternal education, children and adolescent schooling, combined parental educational level)^64-68^, *the employment* (work status, occupational status or profession)^69^, *a composite measure* with more than one SES marker^66^. Sleep was measured with *self-reported items* (by parents most of the time) included in a general survey and measuring subjective symptoms of sleep duration^65,68,69^, sleep quality^64,65,68,69^ and sleep bruxism^65-67^ among children; and *self-reported items based on ICD criteria*^67^.

A detailed analysis of their main conclusions revealed that lower SES was associated with more sleep bruxism, which seems to be the most prevalent sleep disorder in this context. The effect was mediated by sucking behaviour (finger sucking, biting nails or other objects)^66^. Greater prevalence of possible sleep bruxism was observed among adolescents from a higher SES^67^. Students whose mothers had a high level of education were more likely to have low quality of sleep^64^. Lower maternal and adolescent schooling and lower family income were associated with higher sleep duration^68^. Working and higher family income were associated with both short sleep and poor sleep quality^69^. Most studies used subjective measures of sleep variables without objective measures like polysomnography or actigraphy.

### Europe

Similarly, to the USA, European researchers started to investigate influence of SES on children sleep since 1997. Population age in the sixteen studies published from 1997 until 2018 ranged from 1 yr.^30,70^ to 19 yrs.^30,70^. The following SES indicators were measured: *the paternal social class* (white collars or blues collars)^71,72^, *the parental education* (which is one of the most used children SES markers in Europe)^23,26,30,70-80^, *the income* (monthly household income, annual income, net household income per week)^23,30,70^, *the parental material deprivation* (reduced access to material resources, like a car)^30,73^, *the perceived family economy*^75,77^, *a composite measure of SES* (with a combination of more than one indicator)^16,75,81^, *the neighborhood SES* [(measured with the global SES of people living in a specific area, the area SES (measured with the Townsend score) and the family SES (estimated with the parental SES) and housing inadequacy]^16,75,77,80,81^. Among the sixteen studies performed in Europe, the following measures of sleep were employed by the researchers: *self-reported sleep problems* by children, adolescents and their parents^26,29,30,70-74,76,77,79-81^ and *the actigraphy*^16,23^.

Concerning education, findings show that children whose mothers had lower education were more likely to have disturbed sleep^71^. Optimal sleep was positively associated with a high maternal educational level^74^. Higher level of maternal education was negatively related to behavioural induced insufficient sleep syndrome (BIISS)^78^. Habitual snoring was associated with single parenthood or low parental education^81^. Lower parental education was also significantly associated with short time in bed (TIB)^77^, short children sleep^26^ and delayed sleep phase (DSP)^75^. 

Environmental factors (maternal smoking during pregnancy, current parental smoking, electrical cooking, central heating, exposure to road traffic, household pets, number of siblings, nursery care, and breastfeeding >6 months) mediated the effect of parental education^81^. Regarding findings from studies, using a composite index of SES and the neighborhood SES, it is shown that lower SES of parents was associated with increased risk for sleep disorder breathing symptoms of children^80^. Adolescents living in lower socioeconomic conditions experienced significantly poorer sleep outcomes in terms of the timing, duration, consistency, and regularity across the week^16^. The area-based deprivation measure was associated with habitual snoring in white, but not in South Asian children^81^. Average sleep duration was significantly higher for students with high SES by material deprivation^73^. Higher material deprivation, lower maternal education and lower family income were associated with higher odds of reporting at least one sleep problem but neither showed significant effects on sleep duration^30^. Regarding the perceived family economy and the income variable, children with poor family economy had significantly higher odds of reporting difficulties initiating and/or maintaining sleep and short time in bed^77^ and poorer family economy was associated with DSP^75^. Adolescents from families experiencing worsening in their family income reported significantly shorter sleep duration, longer WASO, and lower sleep efficiency than those never having been poor^29^.

### Asia

Twenty-one studies were published from 2000 to 2019. Two of them were cohort studies while the rest were cross sectional. The participant age ranged from 0 to 19 yrs. The following SES measures were used: *the parental education* (categorized as illiterate vs literate; primary school, high school and tertiary education; <12 years vs ≥12 years)^59,82-96^, *the employment status* (classified as employed vs unemployed; employed part-time, on maternity leave, homemaker/at-home parent, student, unemployed/in-between jobs or others)^59,83-85,88,89,92,95,97,98^, *the income* (monthly family income; household annual income, regional per capita gross domestic product)^87,89-93,97,99^, *the questionnaire 4-item family affluence scale* (divided in 3 categories)^100^, the *self-reported perceived SES* (classified better than others, similar to others, poorer than others)^101^ and *a composite SES index* (mixing socioeconomic indicators like occupation and educational level of parents, house ownership, number of rooms in the house, presence of various electrical devices in the house, land value of the house determined according to its location in the city, car ownership possessing, school type, home type)^88,98^. Regarding sleep, the following measures were found: *self-reported or parent-reported sleep duration* (often with the brief infant sleep questionnaire)^83,87,90,95,97-101^, *the self-reported time in bed*^94^, *the actigraphy* (sleep onset, time in bed, sleep efficiency, number of awakenings)^82^, *the insomnia problems* (single question from the WHO - global school based student health survey questionnaire) or *insomnia symptoms* (insomnia self-assessment inventory, often/always vs never/seldom)^88,89,91^, *the parent-reported sleep behaviour* (nightmare frequency, sleep delays, enuresis at night, fear of sleeping alone or darkness)^84-86,92^, *the parent-reported habitual snoring* (more than half the time while sleeping; almost, always/frequently vs occasionally/never in a single question from the children’s sleep habits questionnaire)^93,96^ and *the OSA (obstructive sleep apnea) risk* (score >0.33 in the sleep-related breathing disorder scale of the pediatric sleep questionnaire)^59^.

Children whose mothers had a higher education had longer sleep duration compared to children whose mothers had a lower educational level^83^. The study that used actigraphy as a sleep measure reported that higher parental education level was associated with improved sleep quality (higher sleep percentage and less night waking)^82^. Moreover, adolescents who considered themselves to be “poorer than others” in SES were more likely to report sleep deprivation^101^. Odds for insomnia were higher in students with lower paternal education level and lower SES compared to high SES^88,91^. However many other studies have concluded that children whose parents had higher education levels, the highest income, and in general a higher SES, had a more decreased sleep duration than children with a lower SES^87,98-100^. One of these studies reported that this relation was significantly mediated by screen time (low SES children reported more screen time and thus less sleep time) and academic work (high SES children reported more academic work and thus less sleep time)^100^. In the same manner, lower paternal education was associated with longer weekday time in bed over time^94^, and children whose mothers had a part-time job or were not employed had longer sleep duration^95^. Concerning sleep behaviour problems, lower family income was significantly associated with more frequent nightmares^92^, and lower parental education was associated with presence of nocturnal enuresis in children^84,85^. On the other hand, one study concluded that higher parental education was associated with more sleep behaviour problems (such as sleep delays, enuresis at night, fear of sleeping alone or darkness)^86^. With regard to snoring, the outcomes were different; indeed, one study showed that lower family income and lower paternal educational level were independent predictors of habitual snoring^93^, while another study reported that parental education of a university degree and above was a protecting factor for snoring^96^; otherwise, maternal employment was associated with OSA risk^59^.

### Oceania

Four cross-sectional studies were made from 2012 to 2017, three in Australia and one in New Zealand. The participant ages ranged from 0 to 16 yrs. Researchers used the four following indicators as their main SES measures: the parental education (6 levels)^60^, *the household income* (5 levels)^60^, *the area index* (based on postal codes)^102,103^ and *a composite variable* (family annual income, years of parental education, parental occupational status), used in one study^104^ three sleep measures were found in these studies: s*elf-reported or parent-reported sleep duration*^102,103^, *the parent-reported sleep problems* (difficulty getting to sleep, not happy sleeping alone, waking during the night, and restless sleep)^104^, *the parent-reported sleep latency, sleep duration, and sleep problems* (using children’s sleep habits questionnaire score)^60^.

One of these studies found that there was no difference in sleep duration adherence (adherence to recommendation according to age) between high and low SES children^103^. The others concluded that lower SES was associated with increased odds for parent-reported sleep problems^104^ and that children from low SES areas reported later bedtimes and reduced sleep opportunity than children from higher SES areas^102^. In addition, parents with higher education reported shorter weekday sleep latencies and parents with higher annual income were more likely to report shorter sleep latencies and fewer sleep problems^60^.

### Africa

Finally, there is a cross-sectional study made in Nigeria in 2018. The participants mostly ranged from 10 to 19 years of age. Social class was used as the SES indicator. It was a composite score of parental educational level and occupational status, graded in five levels. Sleep measure was based on *self-reported sleep duration*. The main outcome of this study showed that the odds of having sufficient sleep on the weekdays increased almost 3-fold among the lower social class compared to those of the higher social class^105^.

## DISCUSSION

### Differences in socioeconomic status operationalization: benefits and inconveniences

According to the literature on the topic, socioeconomic factors do indeed influence children and adolescents’ sleep. Nevertheless, the huge amount of measures and conceptualization of SES in the world makes it difficult to compare results. The different methods used to assess SES of children and adolescents are also involved in the heterogeneity of sleep disorder outcomes in this same population. For example, in the USA, several researchers used to include ethnicity/race in their conceptualizations of SES, regardless of the population or the sleep disorders. It was not systematic for SES measures used in articles performed in Canada, in South and Central America, or in Europe across the same period regarding children and adolescents’ sleep. This may be due to the social construction of some countries where being an immigrant from Africa, Latin America or Arabic country is associated with poor standards of living, more stressful life and a lower SES compared to white people or Caucasian individual^20,45,53,106^. This implicit bias/systematic injustice negatively affects people’s perception of ethnic minorities and increases health disparities^2^. Thus, the conceptualization of SES might be biased by this inherent cultural stigma, which brings forth questions about the accuracy of the conclusions made in the literature. This is further supported by the SES measures available in the USA literature, which amplify negative perceptions of race; income-to needs ratio^34,35,38,43,45^, or the “ratio of income to poverty”^36^. Ethnicity is an interesting indicator of SES, but constantly associating non-Caucasian populations with lower SES might be detrimental to interracial relations, especially seeing as a few American authors demonstrated that family SES and family income are not necessarily associated with good sleep in Caucasians^40,41,50,54,57,58^. Similar results were found recently by Plancoulaine et al. (2018)^70^ with a cohort of 1,205 children recruited at birth in France and followed for 6 yrs. They showed that different trajectories of children sleep disorders exist and are influenced by the global lifestyles of parents^70^. Regardless of ethnicity, socioeconomic factors similarly affect children and adolescents’ sleep around the world.

Regarding education, the maternal or paternal educational level alone is not a good measure anymore within our contemporary world, where both parents work and contribute to family income; and recent studies confirmed this by using a composite measure of parental education^20,23,44,60,66,70,99^. Even if equivalent degrees have different names or measurements (for example bachelor in North America is equivalent to license in Europe and Africa), findings are similar and easily comparable; as shown by Manyanga et al. (2018)^23^ in their study performed in 12 countries over 5 continents using income and educational level as SES measures. The indicators “employment” and “income” follow the same trends in terms of viability and comparison; and can be easily standardized and categorized between countries for research purposes^23^.

Finally, researchers in the USA used plenty of new and original SES measures integrating multiple approaches, like the self-reported family’s standard of living^51^, the family economic hardship^56^, the perceived economic well-being^45^ or the subjective social status scale-youth version^49^. This expresses the input of social epidemiology in the populational health research and the evolution of interdisciplinarity in research. These new instruments supply subjective and objective data in addition to measuring new parameters not often considered before (such as the family’s capacity to save money and SES neighbourhoods)^48,81,97^.

### Measures of children’s and adolescent’s sleep

Sleep medicine is a field with clinical guidelines published by medical societies and researchers^107,108^. It is to be expected that there is a homogeneity and a kind of standardization of sleep measures among empirical research published across the world. In North, South and Central America, Australia, Asia, Europe, and Africa, most researchers used self-reported assessment of sleep disorders among children and adolescents. Nevertheless, these subjective measures are often combined with actigraphy and validated questionnaires in North America, Europe, and Asia^16,23,34^, probably because researchers have more funding or opportunities to pay/access some portable/mobile actigraphy systems compared with researchers from Africa or South America. Interestingly, among all these studies, none included polysomnography in their methods, probably due to difficulties to include children in research design requiring a few nights of polysomnographic recordings. Children and adolescents’ sleep seem to be well monitored regardless of the hypothetical influence of SES indicators. However, if SES composite index and SES operationalization are not precisely defined, the results and conclusions have a high probability of being inaccurate. Another weakness in the current literature is the little number of studies which controlled (even not thoroughly) the risk factors for children sleep disorders like cardiovascular diseases risk factors (i.e., obesity or IMC)^50^, behavioural problems (i.e., enuresis and poor sleep hygiene)^53,72,78,84-86^ and breathing disorder symptoms^46,80,81,93,96^, which have a direct or an indirect impact on children’s sleep. 

This paper revealed the existence of an unequal research focus for some children and adolescent sleep disorders. Among the 76 studies, 4 papers investigated excessive daytime sleepiness^35,43,49,78^, 2 papers investigated sleep bruxism^66,67^, 3 articles specifically assessed insomnia symptoms^88,89,91^, 1 article assessed obstructive sleep apnea^59^, 2 papers assessed nightmares/night terrors^65,92^, 8 studies analysed children and adolescents sleep quality (regardless of its origin)^20,28,30,42,49,64,69,82^, and finally 15 articles assessed sleep duration with different approaches regardless of comorbidities and other risk factors^20,23,29,34,36,37,48,70,73,95,97,99,101,105^. There are no studies on somnambulism, narcolepsy and hypersomnia. Some sleep disorders like hypersomnia are more prevalent in adults and old people^107^, which can be a possible explanation for the poor literature of some sleep disorders.

This systematic review sheds light on the poor research productivity of African researchers. Reasons are numerous, such as lack of funding, the quality of facilities, the absence of promotion and governmental strategies regarding local scientific community, the poor research training, the lack of young qualified brains, the weak visibility and the cultural disinterest for research^109^, and probably many other political and contextual reasons^110^ far from the understanding of a foreign observer and out of the control of national politics.

### Recommendations and perspectives for future research

One lesson here is the necessity to unify the definitions of SES across the world and among different research fields. It is impossible to understand each other if we are not talking about the same thing. Even if in theory, it seems similar, in practice there are too many conceptualizations and operationalizations of SES. Of course, it is not enough to just consider education and income to capture parents or children’s SES because of many other influences at a macro, micro and meso level^2^; specifically with sleep disorders, which have many correlations with environment and lifestyle as well as biological homeostasis^111-114^. An idea is to choose SES indicators that are common to all countries or to create a simple language for all researchers and all domains. As a proposal, the 3 followings SES markers can be used (as an example):

- *The worth*: based on few recommendations from OECD framework and several other methods to compute income and its numerous affiliated variables^115-118^; an easier method may be a sum of the annual income (the currency is easy to convert) of the individual (or his parents) and the annual expenses made by the individual (or his parents), with a formula such as $W\left(\mathrm{worth}\right)=\mathrm{AI}\left(\mathrm{annual}\;\mathrm{income}\right)-\mathrm{AE}\left(\mathrm{annual}\;\exp\mathrm{ense}\right)$ . Then, considering the results, categories may be created wherein W<0 represents low income, W=0 represents middle income and W>0 represents high income. Obviously, subcategories would be added^119^. The same rationale may be done with economy, monthly income and monthly expense, etc. Among all the existing ways to calculate the worth^115-118^, social epidemiologists, and economists will find the best formula for all.
- *The education*: nowadays, women are as educated as men, if not more. This means that measures such as maternal/paternal education, number of years of education and highest diploma in the couple; should be reconsidered^120^. A score calculating and taking in account the educational level of one parent or both parents may be developed^121^. For example, primary degree may represent a score of 0, high school degree is 1, undergraduate diploma is 2, master’s degree is 3, PhD/MBA will be 4 and postdoctorate 5. Whatever number of years of education someone has or whatever the degree’s name in a country, it can be matched inside this scale. In the same order of thought, diplomas can be summed for both parents to obtain a global score like $E=\mathrm{PD}\left(\mathrm{paternal}\;\mathrm{diploma}\right)+\mathrm{MD}\left(\mathrm{maternal}\;\mathrm{diploma}\right)$ . As an example, *it can be E = paternal high school degree + maternal master’s degree = 1+3*.
- *The occupational status*: like education, it may be used worker = 1, retired = 2, without employment/on social support = 0. A global score may be computed for one or both parents^119^.

Another lesson is the globalization of research, as shown by the recent interest of Latin America, Oceania, and Africa for the influence of SES on health^109^. More studies performed in these countries by researchers on site will probably enlarge our global knowledge. This contributes in a better way to the local knowledge, compared with the usual inference and appropriation of results published in other countries with different realities^110^.

Sleep disorders are still in study and researchers are using the same guidelines everywhere, with fewer variations than SES. Many mechanisms are still unknown, but methods and language are almost the same as demonstrated by findings above. In conclusion, regardless of the disease or physiological mechanism under investigation, in this case being children and adolescents’ sleep, if SES conceptualization and operationalization are not standardized, their association or impact will never be appropriately understood^122^. A concept that is not understood cannot be associated with quantitative events. Accordingly, current available data in literature might as well be extrapolations of different theories since the understanding of the influence of SES is still mitigated.

## Figures and Tables

**Figure 2:**
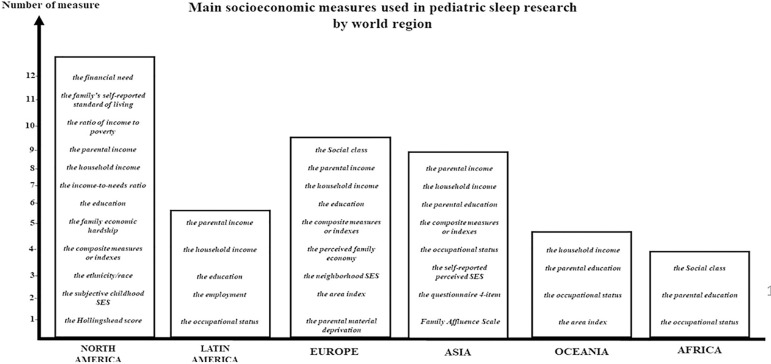
SES indicators employed in pediatric sleep research. In the last decades, there are recurrent type and number of SES indicators which are used by researchers depending on the world region.

**Figure 3:**
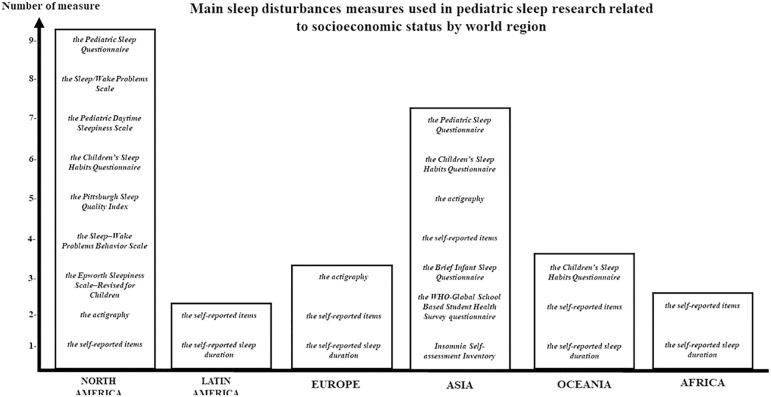
Sleep measures employed in studies assessing the relation SES-SD. In the last decades, there are recurrent sleep measures used by researchers according the world region.
